# Tumour heterogeneity and immune-modulation

**DOI:** 10.1016/j.coph.2013.04.006

**Published:** 2013-08

**Authors:** Mariam Jamal-Hanjani, Eirini Thanopoulou, Karl S Peggs, Sergio A Quezada, Charles Swanton

**Affiliations:** 1 Translational Cancer Therapeutics Laboratory, Cancer Research UK, London Research Institute, London WC2A 3LY, UK; 2 Breast Oncology Unit, The Royal Marsden Hospital, Downs Road, Sutton, Surrey SM2 5PT, UK; 3 UCL Cancer Institute, Paul O’Gorman Building, Huntley Street, London WC1E 6BT, UK

## Abstract

Recent advances in sequencing technologies have revealed extensive intratumour heterogeneity (ITH) both within individual tumours and between primary and metastatic tumours for different cancer types. Such genetic diversity may have clinical implications for both cancer diagnosis and treatment with increasing evidence linking ITH and therapeutic resistance. Nonetheless, whilst limiting the activity of targeted agents, tumour genetic heterogeneity may provide a new therapeutic opportunity through generation of neo-antigens that could be recognised and targeted by the patient's own immune system in response to immune-modulatory therapies. Longitudinal genomic studies assessing tumour clonal architecture and its correlation with the underlying immune response to cancer in each particular patient are needed to follow tumour evolutionary dynamics over time and through therapy, in order to further understand the mechanisms behind drug resistance and to inform the development of new combinatorial therapeutic strategies.

## Highlights

• Intratumour heterogeneity (ITH) has been demonstrated in various tumour types.
• Distinct clonal subpopulations can exist within different regions of a tumour.
• ITH has evident implications for cancer diagnosis and treatment.
• There is increasing evidence for the association between ITH and drug resistance.
• ITH may allow the effective use of immunotherapeutics against tumour neo-antigens.

**Current Opinion in Pharmacology** 2013, **13**:497–503

This review comes from a themed issue on **Cancer**

Edited by **Massimo Santoro** and **Francesca Carlomagno**

For a complete overview see the Issue and the Editorial

Available online 7th May 2013

1471-4892/$ – see front matter, © 2013 Elsevier Ltd. All rights reserved.

**http://dx.doi.org/10.1016/j.coph.2013.04.006**

## Introduction

The existence of distinct subpopulations of cancer cells within a tumour harbouring different behavioural phenotypes, including tumourigenicity, ability to metastasise and evolve resistance to treatment, has been recognised for many years [1]. Recent advances in sequencing technology have given genetic insight into the extent of intratumour heterogeneity (ITH) (for review see [2]), and have contributed to the opinion that ITH is not simply a tumour characteristic, but through the resolution of distinct subclones, may also have the potential to forecast risk of tumour progression and therapeutic outcome. The pattern of genomic instability, and therefore ITH, in tumours can be generated by different processes indicative of clinical outcome. Chromosomal instability (CIN), an initiator of ITH, is associated with poor prognosis in several tumour types [3–5]. Conversely, microsatellite instability (MSI), also a driver of ITH, is associated with good prognosis in colorectal cancers [6]. Therefore, the relationship between ITH and outcome is likely to be complex and dependent not only on the mechanisms generating ITH in individual tumours but also on tumour extrinsic factors such as the potential indirect impact that different forms of ITH may have on the host immune response [7^••^].

**Figure 1:**
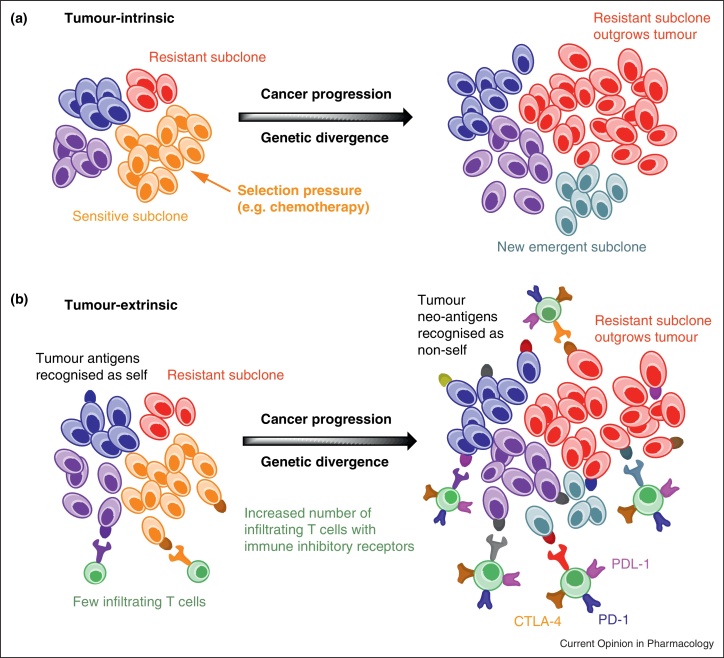
**(a)** Tumour-intrinsic representation of clonal evolution with eventual outgrowth of resistant subclones due to selection pressures, such as cancer treatment, and the emergence of new subclones with continued tumour progression. **(b)** Tumour-extrinsic representation of potential immunological aspects of clonal evolution. With continued clonal evolution, there is the potential for a broader repertoire of tumour-associated neo-antigens recognised as non-self leading to increased T cell infiltration with higher T cell receptor binding affinity. As a consequence, the expression of immune inhibitory receptors, such as PD-1, PDL-1 and CTLA-4, may also be higher. Antibody blockade of such receptors may allow therapeutic intervention that takes advantage of such neo-antigen heterogeneity within a tumour.

In this article we review the clinical implications of ITH for the genetic stratification of tumours, the emerging evidence that suggests the need to investigate the changing nature of tumour subclonal architecture through therapy and the potential impact of such diversity on anti-tumour immunity. We argue that an in-depth understanding of tumour evolution over time, the mechanisms driving tumour diversity and its impact on immunity may lead to the improved management of cancer patients (Figure 1).

## Intratumour heterogeneity and clonal evolution

Phenotypic heterogeneity observed in tumours results from both genetic and non-genetic causes of heterogeneity. Spontaneous tumours are known to arise through Darwinian-like somatic clonal evolution involving the acquisition of ‘driver’ events, such as genetic mutations or copy number variations, believed to affect cancer cell proliferation or survival, along with ‘passenger’ events, assumed to be phenotypically silent without a selective fitness advantage [8]. Non-genetic causes of heterogeneity include epigenetic changes [9], differentiation hierarchies as a result of cancer stem cells [10], stochastic biochemical processes within individual cells and heterogeneous tumour microenvironments [8]. Processes of genetic diversification promote tumour progression through clonal evolution so that tumours appear to be composed of evolving cell populations. The linear model of somatic tumour evolution is that of clonal succession, where a series of clonal expansions are triggered by the acquisition of driver events conferring fitness gain, outcompeting and outgrowing other clones [11]. This model implies that tumours are homogenous for functionally significant mutations, and whilst some tumours are found to evolve through linear steps [12^••^], there is increasing evidence for the existence of genetically distinct clonal subpopulations with substantial genetic divergence coexisting within different regions of the same primary tumour, between primary and secondary tumours, and within metastases [13^••^]. An alternative model of cancer evolution, distinct from the stepwise accumulation of somatic genetic alterations, is that of chromothripsis, in which a cataclysmic one-off genomic event causes massive DNA alterations acting as a driving force for cancer development and progression [14].

## Evidence for intratumour heterogeneity

Recent advances in massively parallel sequencing technologies have enabled the analysis of the complex clonal architecture of both primary and metastatic tumours [15]. Patterns of clonal composition indicate tumour evolutionary paths that underlie tumour progression. An understanding of such evolutionary dynamics is essential in deciphering the clonal origins of metastases and therefore the metastatic process in general, as well as eliciting the mechanisms underlying therapeutic resistance. Several studies have demonstrated genetic diversity within tumours and inferred tumour progression by comparing the mutations and clonal composition between primary and metastatic tumours in different cancer types, including breast, renal, pancreatic, brain and ovarian (for a review see [2,16,17^•^]).

## Intratumour heterogeneity and clinical diagnosis

The validation of predictive biomarkers may be simpler and less subject to tumour sampling bias when present in all regions of a tumour and sustained during disease progression. However, ITH for the expression of genetic and phenotypic biomarkers has been shown in several tumour types. In breast cancer, the amplification of *HER2* predicts response to trastuzumab but its distribution can be heterogeneous in primary tumours and associated with shorter disease-free survival times compared to patients with homogenous *HER2* amplification [18,19]. Yoon *et al*. [20] showed that heterogeneous *HER2* amplification in oesophageal adenocarcinoma independently predicted worse disease-specific survival and overall survival compared to non-heterogeneous *HER2* amplified tumours.

Primary and metastatic tumours can evolve independently and acquire different phenotypes leading to significant genetic divergence, and therefore discordance, between primary and metastatic tumours in terms of biomarkers detected in the diagnostic biopsy [21]. In non-small cell lung cancer (NSCLC), activating mutations in *EGFR* predict response to gefitinib, but discordance for the *EGFR* mutation has been shown between primary and metastatic tumours [22,23]. In primary gastric cancers, heterogeneity of *HER2* amplification and HER2 protein overexpression has been shown within the same tumour, and between diagnostic biopsies and resected tumours [24]. Discordance in *HER2* amplification between primary and metastatic tumours has also been shown in breast cancer [25,26]. In colorectal cancer, Vakiani *et al*. [27] found mutational concordance between primary and metastatic tumours for *KRAS, NRAS, BRAF, PIK3CA* and *TP53* genes. However, in patients with a history of more than one colorectal primary tumour and interval treatment, there was evidence for discordance in *TP53*. These examples demonstrate that relying on a single tumour biopsy may lead to sampling bias in some cases and risk missing potentially therapeutically relevant lesions or contribute to the allocation of a mutation as actionable without establishing clonal dominance [28]. Furthermore, distinct subclonal populations appear to be unequally distributed over space and time, indicating that existing biomarkers are subject to change during disease progression [29]. This may pose a challenge for therapeutic strategies if chosen based on an archival primary tumour biopsy.

## Intratumour heterogeneity and therapeutic outcome

Most advanced cancers still remain incurable despite significant progress in the fields of cancer research and therapy. Response to therapy is generally of limited duration. This may be due to the inevitable evolution and proliferation of resistant subclonal populations, which may exist before the onset of treatment, under the selective pressure of therapies [30,31^••^]. In NSCLC, resistance to the EGFR TKI gefitinib is associated with the positive selection of cells harbouring the gatekeeper *T790M* mutation known to confer insensitivity to gefitinib [32^••^]. Su *et al*. [33] demonstrated that in patients with *EGFR* mutations treated with EGFR TKIs, the presence of low frequency subclones harbouring *T790M* mutations before the onset of treatment was associated with shorter progression-free survival, and Turke *et al*. [34^•^] showed that the presence of subclones with *MET* amplification was associated with EGFR TKI resistance. In colorectal cancer, wild-type *KRAS* predicts sensitivity to anti-EGFR antibody therapies such as panitumumab. Diaz *et al*. [31^••^] showed that by monitoring circulating tumour DNA in patients treated with panitumumab for initially *KRAS* wild-type tumours, the emergence of mutations in *KRAS* could be detected during the course of therapy resulting in acquired resistance. They concluded that subclonal populations harbouring *KRAS* mutations existed before commencing treatment, and that under the selective pressure of anti-EGFR blockade, resistant subclones rapidly expand and repopulate the tumour. In chronic myeloid leukaemia and gastrointestinal tumours, resistance to imatinib due to mutations in the BCR-ABL fusion protein [35] and KIT [36] respectively, has also been demonstrated in the context of clonal evolution. It should be noted that not all cases of therapeutic resistance are necessarily the result of genetic heterogeneity and that non-genetic causes, such as stochastic epigenetic heterogeneity, may also allow the emergence of resistant clones under selection [37]. These examples demonstrate that relapsed clones in metastatic tumours can often be traced back to low frequency subclones before the start of treatment, hence indicating that the extent of ITH is a likely important determinant of therapeutic outcome.

In light of increasing evidence in support of ITH and its role in treatment resistance, there is a need for alternative therapeutic approaches. Gillies *et al*. [38] argue that subclonal populations that respond to initial therapy pass through an evolutionary bottleneck rendering them highly susceptible to a second therapy [39^••^], and that drug resistance in this instance, and the choice of this second therapy, could be anticipated. For example, combined therapy in *EGFR* mutant NSCLC with an EGFR TKI and EGFR-specific antibody could prevent resistance associated with the expansion of a subclone harbouring a *T790M* mutation. Approaches like this would require the development of biomarkers predicting likely resistance mechanisms in different patients, and such mechanisms could be targeted either in combination, or alternating, with standard treatment regimens [40]. Treatment dosing schedules could be adapted to prolong the suppression of resistant subpopulations, for example, drug holidays in androgen-dependent prostate cancer [41] and melanoma [42]. Other adaptive approaches could involve combining standard treatment regimens with drugs targeting phenotypes known to contribute to tumour heterogeneity, such as altered tumour vasculature and altered glucose metabolism [38].

## Intratumour heterogeneity and anti-tumour immunity

Whilst emerging evidence supports the notion that ITH limits the efficacy of conventional and targeted therapeutics, its overall effect on the immune response to cancer may still be of potential benefit for the patient since intratumoural mutational diversity can provide neo-antigens that may be perceived by the immune system as non-self, producing unique opportunities for the generation of anti-tumour immunity. The wealth of data now being generated through whole genome sequencing of tumour samples provides further support for this concept. In silico-based computer algorithms combined with high-throughput *post hoc* analyses of data originally generated by Sjoblom *et al*. [43] revealed that a significant number of candidate tumour neo-antigens arise as a consequence of the multiple gene mutations occurring in breast and colorectal cancers [44]. Furthermore, previous studies in colorectal cancers have shown an association between MSI and good clinical outcomes [6]. MSI is caused by defects in the DNA mismatch-repair system leading to progressive accumulation of mutations, in particular frame-shift mutations that could positively impact immunity. In keeping with this, colorectal tumours with MSI have distinct pathological features, including increased tumour-infiltrating lymphocytes, which have also been associated with better prognosis [45]. One potential explanation is the greater mutational load in tumours with MSI in comparison to CIN tumours, which could result in a higher load of mutated self-peptides or neo-antigens seen as non-self by the immune system, increasing tumour immunogenicity and promoting enhanced T-cell activation and tumour infiltration [46]. Based on this, conventional or targeted agents capable of inducing substantial tumour cell death might produce an *in vivo* ‘vaccine’ or priming effect which could be further enhanced by interference with immune-modulatory pathways. Whilst the neo-antigenic repertoire generated by ITH could be seen as non-self by the immune system, the type of tumour cell death and inflammatory environment within the tumour will define their immunogenicity and the final outcome of the immune response (i.e. tumour progression versus regression). Importantly, immunity to tumour-associated antigens can be potentiated given proper identification and manipulation of immune-regulatory checkpoints restricting T cell function [47,48]. This has been recently illustrated by several high profile clinical trials in which antibody blockade of the immune inhibitory receptors PD-1, PD-L1 or CTLA-4 produced significant clinical benefits against a variety of cancers, including metastatic melanoma [49–52]. In addition to CTLA-4 and PD-1, a large number of trials are currently investigating the anti-tumour activity of monoclonal antibodies against related inhibitory receptors (Lag-3 and B7-H3), as well as of agonistic antibodies against immune-stimulatory receptors. In this particular group, antibodies against different members of the tumour necrosis receptor (TNFR) family (such as OX40, GITR, CD40, CD27 and 4-1BB) are under active investigation either as single agents or in combination with chemotherapies and targeted-therapies (for a review see [53^•^]).

## Future directions and conclusion

Although ITH may complicate diagnostic and treatment decisions, it can be clinically useful in predicting clinical outcome. In Barrett's oesophagus [54^•^] and breast cancer [55,56], ITH has been shown to predict invasive progression. Conversely, extreme CIN, an initiator of ITH, has been shown to be associated with improved long-term survival in oestrogen receptor (ER)-negative breast cancer [57^•^,58]. Extensive ITH in tumours provides greater opportunity for adaptive responses to selective pressures such as hypoxia, chemotherapy and radiotherapy [39^••^] and therefore, measurements of genetic and phenotypic heterogeneity may be of significant value in patient risk stratification [59]. Reliable methods to interrogate tumours and elicit their underlying clonal architecture need to be developed in order to test the association between distinct mechanisms of ITH and clinical outcome. ITH poses a challenge for effective cancer therapy, and the resulting heterogeneous expression of biomarkers may have implications in terms of accurate diagnosis and treatment outcome [17^•^]. Longitudinal genomic analysis of tumours at diagnosis, during treatment and at relapse may inform new approaches and shine a light upon tumour adaptive mechanisms through therapy. Clinical trials and biomarker studies should consider such designs to demonstrate the potential benefit of adaptive therapy in response to tumour evolution through the disease course. Obtaining multiple tumour biopsies to study tumour clonal architecture in such trials may be clinically challenging but should at least be considered. Potential non-invasive alternatives to re-biopsy in patients with multiple or inaccessible metastases may include molecular imaging and circulating tumour DNA [60].

With the development of improved technologies allowing the interrogation of ITH, our understanding of tumours and their evolutionary trajectories may lead to better design of clinical trials in search of improved therapeutic interventions to anticipate the emergence of drug resistance mechanisms and generate improved predictive and prognostic biomarkers [61,62]. Whilst cancer cells cannot anticipate future evolutionary events or the selective pressures they may encounter, we should prepare for, and proactively manage, such changes and use our acquired knowledge of tumour evolutionary dynamics to predict and guide treatment strategies in order to attempt to improve patient outcomes. Underlying mechanisms of ITH that result in increased mutational diversity may in theory result in the generation of neo-antigens recognised by the immune system as non-self. The pipeline of new immunotherapeutic drugs offers a newer and larger window of opportunity through which tumour sensitivity could be enhanced via the rational combination of targeted and immune-therapies where targeted therapies will promote tumour destruction and neo-antigen exposure (generated through ITH) to the immune system, whilst manipulation of immune-regulatory pathways will potentially enable a powerful, diverse and durable response against the tumour.

## Conflict of interest

None declared.

## Role of the funding source

None.

## References and recommended reading

Papers of particular interest, published within the period of review, have been highlighted as:

• of special interest
•• of outstanding interest
